# Rapid recovery from near-fatal immune reconstitution inflammatory syndrome and acute respiratory distress syndrome following neutropenic recovery by cytokine adsorption

**DOI:** 10.1186/s12890-026-04619-y

**Published:** 2026-08-25

**Authors:** Karl Bihlmaier, Laura Vonbrunn, Axel Schmid, Larissa Herbst, Krystyna Bujok, Felix A. Büstgens, Rebecca Wätzig, Heidi Waibel, Mario Schiffer, Carsten Willam

**Affiliations:** 1 Department of Internal Medicine 4, Nephrology and Hypertension, Friedrich-Alexander University Erlangen-Nürnberg (FAU) and University Hospital Erlangen, Ulmenweg 18, 91054 Erlangen, Germany; 2 Institute of Radiology, Friedrich-Alexander University Erlangen-Nürnberg (FAU) and University Hospital Erlangen, Maximiliansplatz 3, 91054 Erlangen, Germany; 3 Department of Internal Medicine 5, Hematology and Oncology, Friedrich-Alexander University Erlangen-Nürnberg (FAU) and University Hospital Erlangen, Ulmenweg 18, 91054 Erlangen, Germany; 4 Present Address: Emergency and Acute Medicine, Klinikum Fürth, Jakob-Henle-Straße 1, 90766 Fürth, Germany

**Keywords:** Neutropenia, Immune reconstitution inflammatory syndrome, Acute respiratory distress syndrome, Cytokine adsorption

## Abstract

**Background:**

In the presence of clinical or microbiological evidence of pneumonia, emerging leukocytes in recovery from neutropenia undergo substantial migration to the site of infection, where they elicit immunogenic reactions. The administration of granulocyte colony-stimulating factors is a risk factor for dysregulation of this immune response, which can lead to ARDS. However, there is a paucity of knowledge regarding the severe forms of near-fatal IRIS.

**Case presentation:**

This case report describes the successful treatment of a hematologic patient with life-threatening *Immune Reconstitution Inflammatory Syndrome* after recovery from neutropenia and a subsequent acute respiratory distress syndrome involving cytokine adsorption. The present report delineates the case of a 65-year-old male afflicted with plasmoblastic lymphoma and neutropenic sepsis with pneumonia. *Escherichia coli* was identified in bronchoalveolar lavage fluids, and the patient was successfully treated with piperacillin and tazobactam. After recovering from neutropenia, the patient's respiratory function abruptly worsened, resulting in a secondary, severe ARDS. This deterioration was accompanied by increasing interleukin-6 levels. Therapeutic approaches as prone positioning or volume depletion using renal replacement therapy with high ultrafiltration rates did not lead to a significant respiratory improvement. Initiation of cytokine adsorption resulted in an immediate and absolute respiratory recovery.

**Conclusions:**

Immune reconstitution inflammatory syndrome might cause dysregulated pulmonary immune responses following recovery from neutropenia with consecutive severe ARDS. This case report is the first to link IRIS-induced severe ARDS to a hyperinflammatory condition and to the successful therapeutic approach of cytokine adsorption. Ultimately, only cytokine adsorption was able to rapidly improve this suspected IRIS-associated hyperinflammation in an otherwise life-threatening ARDS.

## Background

*Immune reconstitution inflammatory syndrome* (IRIS) is defined as an excessive inflammatory reaction that occurs during the process of immune system reconstitution. The process is initiated by an excessive immune response during recovery of immune cells, particularly in response to acute or subacute infections [1].

IRIS was first discovered in the 1980 s in patients with tuberculosis (TB) and leprosy receiving treatment [1]. Since the introduction of the first HAART (highly active antiretroviral therapy) drug zidovudine in 1987 and combination therapy in 1996, IRIS emerged as a severe complication in patients infected with the human immunodeficiency virus (HIV) [1]. Approximately 10–25% of HIV patients develop an IRIS after starting HAART [2]. The onset is variable, typically occurring a few days to 6 months after initiation of antiretroviral therapy [3]. HIV patients present a significant increase in CD4 + and CD8 + T cells after the initiation of the antiretroviral therapy. This enhances both a cell-mediated and an antibody-mediated immunity with an excessive, pathogen-specific cellular immune response, increased effector memory T cells, regulatory T cell dysfunction, and increased cytokines and inflammatory serum markers (C-reactive protein, interleukin-6, interleukin-12, or tumor necrosis factor-α) [1, 3, 4].

Risk factors for IRIS in HIV patients include starting HAART treatment in younger or male patients, HAART-naïve patients, low CD4 + T cell counts (less than 100/µl), a high viral load of HIV RNA, a rapid decline of HIV RNA after therapy initiation, and a latent opportunistic infection [2, 3, 5, 6]. Common pathogens associated with the syndrome include mycobacteria, varicella zoster, herpes viruses and cytomegalovirus (CMV) [3, 5]. Eventually, genetic markers may also play a role, particularly in cases of herpes (HLA-A, HLA-B44, or HLA-DR4) and mycobacterial infections (TNFA-308*1, IL6-174*G) [3].

In addition to infections, IRIS occurs in other conditions, including solid organ transplantation, following hematopoietic recovery after chemotherapy against hematological malignancies, the postpartum period, and treatment with tumor necrosis factor antagonists [1, 3]. For neutropenic patients there is only little data available [7–9]. Risk factors for IRIS in hematologic patients are a preexisting clinical or microbiological pneumonia and the use of granulocyte colony-stimulating factors during neutropenia [9, 10].

Severe IRIS involving CNS (cryptococcal meningitis (CM)-associated IRIS, CNS TB-IRIS) or pulmonary infections presents a high mortality rate [3]. The treatment of IRIS includes supportive measures such as volume management and electrolyte balancing, treatment of the underlying pathogen (anti-infective therapy), and in some cases immunomodulatory regimens as e.g. corticosteroids (tuberculosis IRIS, TB-IRIS) or NSAIDs [1, 3, 4, 11].

Blood stream adsorption filters have been developed in order to remove inflammatory cytokines in sepsis [12]. It has been used for the treatment of sepsis, severe pneumonia, ARDS, in cardiac surgery using cardiopulmonary bypass or after cardiac arrest [12]. Various commercially available adsorbers have been developed including CytoSorb® (removal of cytokines), Seraph-100® (removal of cytokines and pathogens), Toraymyxin® (removal of endotoxins), and oXiris®, HA330/380 (removal of cytokines and endotoxins) [13].

## Case presentation

A 65-year-old male patient with febrile neutropenia was transferred to the nephrology intensive care unit (ICU) at our university hospital. His medical history included plasmoblastic lymphoma of the right maxilla, chronic kidney disease (CKD) stage 3b, and arterial hypertension. The week before admission, he underwent his fifth cycle of chemotherapy according to the CHOEP protocol, which consisted of cyclophosphamide 750 mg/m^2^, doxorubicin 50 mg/m^2^, vincristine 2 mg, etoposide phosphate 100 mg/m^2^, and prednisolone 100 mg for five days. The patient was planned for stem cell harvest, therefore granulocyte-colony stimulating factor support was given. While at home, he developed a fever up to 40.1° C in neutropenia (white blood cells, WBC 0.18/nl) and sinus tachycardia at 134 beats per minute. In the emergency room, he presented symptoms of shock with a blood pressure of 86/40 mmHg. We initiated antibiotic therapy with piperacillin and tazobactam (P/T). He received eight liters of crystalloid infusions and was transferred to the ICU due to refractory hypotension, tachycardia, tachypnea, and lactic acidosis (4.7 mmol/l).

Upon admission to the ICU, high-dose vasopressor therapy with norepinephrine at a rate of up to 0.64 µg/min/kg was initiated. With high-flow nasal oxygen therapy (HFNOT) at 50 L per minute and a fraction of inspired oxygen (F_i_O_2_) of 1.0, the partial pressure of arterial oxygen (PaO_2_) was 60 mmHg. Therefore, the patient was intubated and ventilated invasively. He had moderate acute respiratory distress syndrome (ARDS) with a PaO_2_/F_i_O_2_ ratio of 136 mmHg. Bronchoalveolar lavage fluids as well as urine diagnostics revealed *E. coli*, sensitive to piperacillin and tazobactam. Blood cultures remained sterile.

The patient began to recover from pneumonia during the first 48 h of therapy, and the P_a_O_2_/F_i_O_2_ ratio increased to 296 mmHg. Procalcitonin levels improved. According to the patient's chemotherapy protocol, granulocyte colony-stimulating factors (480 µg Filgrastim Zarzio) were administered 20 h post ICU admission to treat neutropenia.

**Fig. 1:**
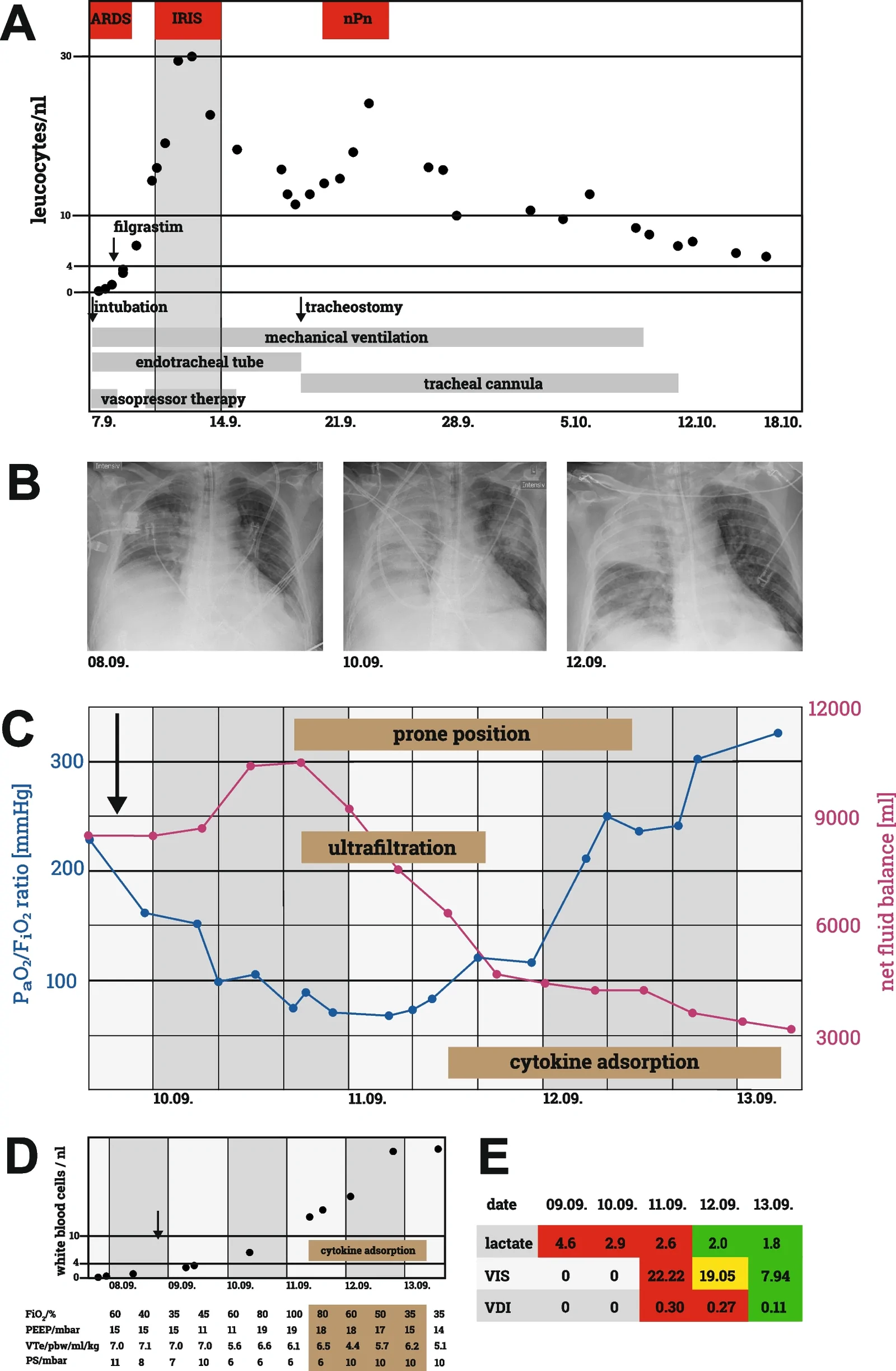
**A** Overview of the ICU treatment. Key elements during the ICU treatment are shown. Red boxes indicate primary ARDS, secondary IRIS, and nosocomial pneumonia (nPn). Development of white blood cells is shown for clinical correlation. The upper arrow indicates injection of filgrastim. Grey boxes indicate need for mechanical ventilation using endotracheal tube or tracheal cannula, respectively. The lower arrows indicate orotracheal intubation and tracheostomy. Vasopressors were used during early ARDS and early IRIS phases. **B** Follow up of X-ray chest radiographs. X-ray chest radiographs were taken every other day in order to document development of opacities correlation to IRIS-induced ARDS. **C** Impact of different therapeutic maneuvers on IRIS-induced ARDS. The PaO₂/F_i_O₂ ratio in the critical period is shown in blue. The arrow indicates the onset of leukocyte recovery starting from total neutropenia. PaO₂/F_i_O₂ ratio worsened from mild to severe ARDS within 18 h. Prone position and a high ultrafiltration rate of 300 ml/h had no effect on the PaO₂/F_i_O₂ ratio. The net fluid balance of the patient is shown in pink. After cytokine adsorption had been initiated, severe ARDS was cleared *in toto* to PaO₂/F_i_O₂ ratios above 300 mmHg. **D** Ventilator settings in correlation to leucocytosis and cytokine adsorption. The graph shows the WBC development from neutropenia to leukocytosis. The table lists ventilator settings. First, ventilator settings could be reduced due to therapy of E. coli ARDS. Second, IRIS re-induced an ARDS. Note that symptoms of IRIS often appear before WBC rises. Third, following cytokine adsorption ventilator settings could be reduced rapidly.**E** Development of serum lactate, vasoactive-inotropic score, and vasopressor dependency index during IRIS and cytokine adsorption. With start of cytokine adsorption, vasoactive-inotropic score (VIS) and vasopressor dependency index (VDI) declined rapidly. Lactate levels normalized

Within 24 h, the patient developed leukocytosis, which peaked at 31.000/µl (Fig. 1A). Subsequently, the patient's respiratory capacity deteriorated rapidly, and he developed severe, secondary ARDS, as evidenced by increasing diffuse opacities on his chest X-ray (Fig. 1B) and a PaO_2_/F_i_O_2_ ratio of 67.8 mmHg (F_i_O_2_ 0.9). The SOFA score was 13. Serum IL-6 levels increased to 2188 pg/ml. Thus, a life-threatening IRIS was suspected.

In order to test for other clinical factors that could have led to severe ARDS, further microbiological and virological diagnostics were carried out. Iterative BALFs were taken. Tests including galactomannan, blood culture samples, serum beta-D-glucan, and PCRs against HSV-1/2 and CMV were repeatedly reported negative, and there were no transfusions during the time of respiratory deterioration that could have led to a transfusion-related acute lung injury (TRALI) or transfusion-associated circulatory overload (TACO). Transthoracic echocardiography and cardiac enzyme profiles showed normal cardiac function, and pleural effusions were ruled out by ultrasound examinations.

Due to worsening of gas exchange, fluid overload was suspected, and the patient was placed in the prone position which was maintained continuously for 28 h. We initiated continuous renal replacement therapy (CRRT) as venovenous hemofiltration connected to a dialysis ring line system with unfractionated heparin as anticoagulation, predilution, a blood flow rate of 200–230 ml/min, and an effluent dose of 14.1 ml/kg/h because of a sepsis-associated-acute kidney injury [14] and declining diuresis. To decrease pulmonary water shift and ensure sufficient oxygenation, we initiated an ultrafiltration rate of 300 ml/h which led to a removal of 5.5 L of fluid in the next 24 h. Nevertheless, the PaO₂/F_i_O₂ ratio showed no significant improvement (Fig. 1C), but the vasopressor dosage increased from 0.1 to 0.28 µg/min/kg. Since fluid removal did not improve pulmonary function and radiographics, we suspected an IRIS-induced ARDS. We determined IL-6 (rising rapidly to 2188 pg/ml), stopped further ultrafiltration and initiated cytokine adsorption. Cytokine adsorption was carried out for a total of 39 h using two CytoSorb 300 adsorbers as tandem therapy during CRRT, for 20 h and 19 h, respectively (Fig. 1C). Within 18 h, the PaO₂/F_i_O₂ ratio improved to 306 mmHg, and ventilator settings could be reduced. F_i_O_2_ was reduced from 1.0 to 0.35. The PEEP could be lowered from 19 to 14 mbar (Fig. 1D). Simultaneously, the vasopressor dosage could be reduced to 0.04 µg/h/kg. Vasoactive-inotropic score (VIS) declined from 22.22 to 7.94 and vasopressor dependency index (VDI) declined from 0.30/mmHg to 0.11/mmHg during cytokine adsorption (Fig. 1E). During the adsorption therapy, lactate levels normalized (Fig. 1E) and IL-6 levels declined to 769 pg/ml.

Therapeutic drug monitoring (TDM) of P/T was conducted after five administrations of P/T (4.5 g q8h), yielding a through level of 62.2 mg/l. Thus, P/T administration was reduced to 4.5 g q12h. To avoid underdosing, administration of P/T was increased back to 4.5 g q8h with start of cytokine adsorption. Procalcitonin and C-reactive protein dropped significantly during this treatment. After cytokine removal was completed, administration was re-adjusted to 4.5 g q12h and finally discontinued two days later after 8 days of total duration of antibiotic therapy.

After 60 h of continuous renal replacement therapy (CRRT) and one additional intermittent hemodialysis session, renal function recovered and the dialysis catheter was removed. Weaning from the respirator was difficult and prolonged due to tertiary ventilator-associated nosocomial pneumonia. The patient received dilatative tracheostomy, and weaning was completed after 24 days. With physiotherapy the patient could walk the corridor. He was discharged to a rehabilitation unit. In the long term, the patient presented in follow-up visits with good health and moderate polyneuropathic symptoms in the distal lower extremities. Although chemotherapy had to be discontinued, no relapse of the plasmoblastic lymphoma occurred until today.

No major adverse events occurred during the cytokine filter treatment. Circuit clotting occurred twice, most likely due to low-dose heparinization.

Hemodynamics improved during treatment (Fig. 1E), and there were no documented phases of hypotension. The platelet count was very low with ICU admission following chemotherapy, but it regenerated during adsorption therapy. There were no significant alterations in coagulation parameters nor any stigmata of bleeding.

## Discussion

IRIS was first described in the 1980 s in patients with TB starting their therapy [1], but was mostly described in HIV patients when treatment with antiretroviral medication started [15], and immune reconstitution led to an excessive immune response.

However, IRIS in hematologic patients with subsequent ARDS symptoms is a repeatedly reported but less well-investigated disease entity. [7–9, 16, 17]. In particular, when neutropenia recovers, ARDS symptoms can occur in the presence of clinically or microbiologically detected pneumonia, even when it is not detectable on a thoracic X-ray imaging [8]. In non-HIV patients, little data exists on severe IRIS-induced ARDS. They consist mainly of case reports and case series.

As leukocytes recover and migrate to the site of pulmonary infection, they might secrete interleukins, resulting in local as well as systemic effects. Thereby, symptoms of IRIS often appear before the increase in WBCs becomes measurable. Conventional therapeutic methods, including anti-infective therapy, reduction of water load, prone positioning, and escalation of mechanical ventilation, failed to improve the patient's progressive respiratory failure. Since interleukins are found to be elevated in HIV-associated IRIS and the administration of corticosteroids is a feasible therapy option in TB-IRIS, we hypothesized an equivalent mechanism in IRIS after recovery from neutropenia [18, 19]. We then discussed various options of immunomodulatory therapy. Immunosuppressive regimens have been previously used in tuberculosis or HIV therapy-associated IRIS [20]. A few cases have been reported of the successful use of corticosteroids in neutropenic patients with invasive pulmonary aspergillosis [16, 21]. However, since IRIS can be considered a temporary overactivation of the immune system, we decided to focus primarily on removing proinflammatory mediators rather than starting immunomodulatory medications such as corticosteroids or tocilizumab.

A similar syndrome that may be considered is the neutrophil engraftment syndrome (NES). NES occurs in neutrophil recovery after hematopoietic stem cell transplantation. Symptoms resemble that of IRIS and consist of fever, rash, pulmonary infiltrates with hypoxemia, encephalopathy, or diarrhea [22, 23]. NES cases requiring treatment are rare [24], and, in contrast to IRIS, NES usually occurs in the context of non-infectious clinical conditions.

In the reported case, applying a cytokine adsorber first led to imminent respiratory and clinical improvement, accompanied by a rapid decrease in IL-6 levels in the blood. Furthermore, the dose of vasopressors could be reduced, and the X-ray opacities disappeared. There were no adverse events.

A strength of our report is the preliminary exclusion of secondary microbiological and cardiogenic effects as well as the clinical proof that fluid overload does not account for the ARDS. Thus, diagnosis of ARDS in IRIS was conclusive though quite rare. A limitation is the singularity of our report.

In principle, mild or moderate IRIS is self-limiting. However, when life-threatening complications occur, such as ARDS or AKI, and when IL-6 levels are elevated, the risk of mortality increases. Since these conditions do not respond to general supportive therapeutic methods, cytokine adsorption can be a lifesaving option. There are no established treatment guidelines regarding the duration of therapy, the blood flow rate, or the number of adsorbers [12]. In theory, the decision to use an adsorber can be based on the patient's immediate clinical condition and the presence of high IL-6 levels. However, blood adsorption can lead to complications, such as those resulting from catheter insertion, thrombocytopenia, and nonspecific removal of hydrophobic plasma components, including anti-inflammatory mediators, hormones, and coagulation factors. [25–27]. Furthermore, antibiotics are also eliminated, which can result in subtherapeutic levels of antibiotics [28]. Thus, aggressive antibiotic TDM and/or prophylactic dose escalation of antibiotics is mandatory during cytokine adsorption. It will be interesting in the future to further prove safety and efficacy of cytokine removal in this life-threatening disease entity.

## Conclusions

IRIS can be a life-threatening condition for patients whose immune cells are recovering from chemotherapy. While leukocytes recover from neutropenia, systemic interleukins might be elevated during respiratory deterioration in suspected IRIS-induced ARDS.

ARDS in IRIS can occur, especially when subclinical pulmonary infections are present. In cases of life-threatening complications, such as ARDS and AKI, immediate cytokine adsorption might help to counteract severe inflammatory storm.

In particular, cytokine adsorption can easily be applied as a bridging instrument when renal replacement therapy is initiated until immune cell recovery is no longer accompanied by a cytokine storm. The potential side effects must be weighed against the potential clinical benefit.

## Data Availability

The data supporting these findings are available from the corresponding author.

## Abbreviations

AKI: Acute kidney injury
ARDS: Acute respiratory distress syndrome
BALF: Bronchoalveolar lavage fluid
CHOEP: Chemotherapy with cyclophosphamide, doxorubicin, vincristine, prednisolone, and etoposide phosphate
CRRT: Continuous renal replacement therapy
HAART: Highly active antiretroviral therapy
HFNOT: High flow nasal oxygen therapy
HIV: Human immunodeficiency virus
ICU: Intensive care unit
IL: Interleukin
IRIS: Immune reconstitution inflammatory syndrome
P/T: Piperacillin/tazobactam
RNA: Ribonucleic acid
SOFA: Sequential organ failure assessment score
TACO: Transfusion-associated circulatory overload
TB: Tuberculosis
TDM: Therapeutic drug monitoring
TNF: Tumor necrosis factor
TRALI: Transfusion-related acute lung injury
VDI: Vasopressor dependency index
VIS: Vasoactive inotropic score
WBC: White blood cells
